# Contrasting Trait-Mediated Mechanisms Shape Peatland Testate Amoeba Communities Under Long-Term Drying Across Fen-Bog Gradient

**DOI:** 10.1007/s00248-026-02796-1

**Published:** 2026-06-03

**Authors:** Brunella Palacios Ganoza, Olivia Kuuri-Riutta, Anna M. Laine, Minna M. Väliranta, Edward A. D. Mitchell, Eeva-Stiina Tuittila

**Affiliations:** 1 School of Forest Sciences, University of Eastern Finland, Joensuu, Finland; 2 Ecosystemes and Environment Research Programme, Environmental Change Research Unit, University of Helsinki, Helsinki, Finland; 3 Laboratory of Soil Biodiversity, University of Neuchâtel, Neuchâtel, Switzerland

**Keywords:** Climate change, Community assembly, Environmental filtering, Functional redundancy, Functional traits, Protist communities

## Abstract

Boreal peatlands store vast amounts of carbon and regulate regional hydrology, making their stability critical under accelerating climate change. This stability depends on community-level resilience and resistance shaped by the functional traits of organisms. Although climate-induced drying is already reshaping peatland communities, the trait-based mechanisms supporting stability in soil biota remain poorly resolved. We assessed stability in testate amoebae (TA) – a key group of the soil food web – by comparing functional trait patterns in ambient control plots with plots subjected to two decades of experimental water level drawdown (WLD) across three peatland types (rich fen, poor fen, and bog). In ambient conditions, null models revealed strengthened environmental filtering along the fertility gradient; nutrient-rich fen showed highest diversity and functional redundancy. Long-term WLD, however, intensified environmental filtering by reshaping the communities across all peatland types. Functional beta diversity revealed contrasting stability mechanisms: rich fen maintained functional stability through resilience, with major species turnover but minimal functional change, whereas bog communities retained function through resistance, relying on drought-adapted traits and showing minimal species turnover. Contrastingly, the poor fen lost functional stability, as low redundancy combined with dominance of wet-adapted traits led to both species and functional turnover. Under moderate drying, bogs are most likely to maintain functional stability, whereas fens, particularly poor fens, are more vulnerable. These trait-mediated differences indicate peatland type-specific functional thresholds with implications for predicting stability and carbon dynamics under future climates. Overall, continued warming increasingly compromises peatland soil biota and the ecosystem functions they mediate.

**Supplementary Information:**

The online version contains supplementary material available at 10.1007/s00248-026-02796-1.

## Introduction

Boreal peatlands are major reservoirs of terrestrial carbon [1] and host specialized biodiversity adapted to persistently wet, acidic, and nutrient-poor conditions [2]. However, they are increasingly threatened as temperatures in high latitudes rapidly rise [3], possibly increasing evapotranspiration beyond compensatory precipitation inputs [4, 5]. Consequent drying can disrupt ecosystem functions, reduce resilience, and compromise the stability of soil processes. While soil biotic communities play a critical role in sustaining ecosystem stability [6], the trait-mediated stability mechanisms that buffer the effects of environmental stress on peatlands’ functioning remain poorly understood. In particular, it is unclear how long-term drying affects the functional stability of soil protists, critical regulators of carbon and nutrient cycling. Addressing this knowledge gap is essential for understanding peatland responses to ongoing climate-driven hydrological shifts.

Theoretically, the mechanisms through which environmental changes influence communities’ functional stability are well established [e.g., 7, 8]. Community assemblage is governed by stochasticity (randomness) and deterministic processes: environmental filtering, where species with traits suited to local conditions are selected [8], and niche differentiation, where species adopt distinct strategies to reduce competitive overlap [7]. Empirical studies, primarily in aboveground communities, have applied trait-based approaches to infer and disentangle how these mechanisms operate under changing climate [e.g., 9, 10]. However, results vary widely across ecosystems and climate scenarios, which makes it difficult to determine when trait-environment relationships have predictable functional outcomes and when they are shaped by context-dependent factors [9]. This variability underscores the need for long-term empirical assessments in systems undergoing directional climate forcing.

Ecological stability – the tendency of communities to withstand and recover from a disturbance – emerges from resilience and resistance processes [11–13]. These two processes are fundamentally mediated by functional traits. Communities may resist disturbances when their traits are not sensitive to the effect, leading to unaltered communities or functions [11–13]. Contrary, resilient communities respond to disturbance but are able to recover. A key stability mediator/mechanism of functional resilience is redundancy, where multiple species perform similar ecological roles, thereby buffering ecosystem functioning against species loss or turnover [14, and references therein]. Therefore, communities with high functional redundancy may be more resilient to disturbance, whereas communities with low redundancy may experience disproportionate functional shifts, potentially altering their ecological role.

Climate-warming-induced drying of peatlands strongly impacts key soil protists, potentially altering the structure and function of peatland microbial food webs [15]. Soil protists have a strong effect on ecosystem functions as they represent the main predators of microbes and directly influence photosynthesis and respiration [15, 16]. In peatlands, testate amoebae (TA) account for the largest fraction of the total protozoan biomass [17] and represent the top predators within the microbial food web [16]. TA are highly sensitive to water table depth, even to the extent that species can be used as valuable palaeohydrological proxies [18] and their traits respond consistently to environmental changes. For example, TA biovolume typically decreases under drying [19], which can reduce their trophic status [20], weaken top-down control on decomposers, and potentially destabilize microbial food webs [6, 21]. Because TA traits reflect ecological strategies shaped by hydrology and climate, they are powerful model organisms for investigating the processes governing soil biodiversity-stability relationship under long-term drying.

This study aimed to assess whether trait-mediated mechanisms in soil protist communities maintain – or fail to maintain – functional stability under climate-induced drying across boreal peatlands. Using a two-decade water level drawdown (WLD) experiment setup, we compared TA communities and functional traits between WLD treatment and ambient control areas across three peatland types. More specifically, we addressed two hypotheses:

- H1: TA communities are strongly affected by altered environmental filtering. We therefore expected size-related traits to show a stronger environmental filtering and niche differentiation under WLD than under ambient conditions.
- H2: TA communities with higher functional redundancy are functionally more stable. Thus, in highly redundant TA communities, we expected WLD-induced species turnover to be decoupled from functional turnover.

## Materials and Methods

### Study Site

We used a long-term WLD experiment started in an eccentric raised peatland complex, Lakkasuo (located in Southern Boreal Finland, 61°47′N, 24°18′E), in 2000–2001. It consists of three experimental WLD areas and corresponding control areas, each pair representing a different peatland type (site): a mesotrophic fen, an oligotrophic fen, and an ombrotrophic bog, here referred to as rich fen, poor fen, and bog, respectively. See further details in [22]. The sampling covers variation representative of boreal peatlands, but as the experiment was conducted within a single peatland complex, we lack true replication across the three sites. Although this limits the generalization of our results, it allows us to reveal the trait-mediated mechanisms driving the biodiversity-stability relationship between control and WLD areas.

Pre-experiment, the control and WLD areas had similar vegetation and water table. Since then, significant changes have occurred in the fen WLD areas, where the development of tree stand has altered abiotic conditions for the understory vegetation [23, 24]. The contrast between treatments is smaller in the bog, where the vegetation and TA communities have changed slightly [23, 25].

### Data Collection

Data have previously been used to assess the use of TA as a proxy for climate-induced drying and associated changes [25; for detailed methodological description].

We collected the upper 3 cm of 3 to 10 *Sphagnum* moss shoots from 8 to 10 samples from each of the six study areas (total = 53) during summer 2022 and stored the samples in 15 ml of 4% formaldehyde. The samples were shaken for two minutes, filtered through a 150 μm sieve, centrifuged, and analyzed by light microscopy at 200x and 400x. We targeted 150 individuals identified to species level [26] using Siemensma [27] and McKeown [28]. For traits, we recorded aperture size, biovolume, trophic status (mixotroph or heterotroph), aperture position, test compression, and test material (Table 1 for definitions and ecological implications). Test length and width and aperture size were microscopically measured, aiming 5 replicates per taxa per sample. Some individuals were unmeasurable (e.g. due to poor placement), but the final dataset represents at least 80% of the community in each sample [32]. For low-count species, we used the mean value calculated from the same study area. Biovolume was calculated using a different formula for each test shape, as in Fournier [31]. Taxon-specific values for aperture position, trophic status, test compression, and material derive from Fournier [30] and supplementary material from Fournier [31], complemented with Siemensma [27].

**Table 1:** Functional traits measured, their response patterns to disturbances, and ecological implications

Trait	Unit	Description	Response to disturbances	Ecological impact	References
Aperture position	Factor	Position of the aperture within the test: axial (1), acrostomic (2), or plagiostomic (3)	Disturbed conditions favor plagiostomic	Related to contribution to the food web	Jassey [29], Fournier [30]
Aperture size	µm	Width of the test aperture	Disturbed conditions favor small apertures	Related to the prey size and food web functioning	Fournier [31]
Biovolume	µm³	Volume of the test occupied by the living amoeba (80%)	Disturbed conditions favor small size	Related to the metabolic rate and the capacity of the food web to process energy	Fournier [30], Reczuga [21]
Mixotrophy	Binary	Presence (1) or not (0) of photosynthetic endosymbionts	Wet and unshaded conditions favor mixotrophy	Related to peatland C cycling and bacterial grazing	Jassey [29]
Test compression	Factor	Spherical (1), sub spherical (2), compressed (3), or strongly compressed (4)	Disturbed conditions favor the compression of the test	Related to the ability to stay active and contribute to the food web in drier conditions	Fournier [30]
Test material	Factor	Test made of protein (1), silica (2), silica + organic (3), calcite (4), recycled idiosomes (5), or xenosomes (6)	Protein is favored by wet and unshaded conditions, silica by dry conditions. The knowledge is controversial for recycled idiosomes, silica + organic, calcite and xenosomes	Related to the availability of material and/or prey to construct the test or build a self-secreted test	Fournier [30], Geisen [16]

### Statistical Analyzes

All statistical analyzes were performed using R version 4.2.2 [33]. Due to varying total counts across the sampling plots and the correlation between total count and the number of species, we standardized the abundance data. We calculated the relative abundances and applied a threshold based on the minimum relative abundance in the plot with the smallest number of observations (< 0.8%). This resulted in a dataset that covered 52 of the 66 initially identified taxa. We calculated the community weighted mean (CWM) functional traits for each plot by weighing the mean trait value of the taxon in the specific community, by their relative abundances.

To examine the variability in TA functional traits between ambient and WLD conditions, we applied a principal component analysis (PCA) using the *vegan* package [34]. As environmental variables, we used peat nutrient contents and pH [23], and *Sphagnum* water content, water table depth, and shading intensity [24].

Additionally, to further examine trait-variation mechanisms, we constructed linear models to quantify the relative contribution of species turnover and intraspecific trait variability to variation in CWM biovolume and aperture size. Following Laine [22], we generated two additional CWM datasets: (1) fixed CWM, calculated using average species-level trait values from the full dataset weighted by their relative abundances, and (2) difference CWM, calculated by subtracting the fixed CWM values from the observed CWMs. For each trait, we fitted three linear models (observed CWM, fixed values, or difference), using site fertility and treatment as predictors. Site fertility gradient was represented by the PC1 axis of a previous PCA of environmental variables in the study areas [24]. From each model, we extracted the sum of squares (SS) to calculate the SS cov. (species turnover and intraspecific trait covariation) by subtracting SS fixed and SS difference from SS observed [35]. Percentages of variability contributed by each component (species turnover, intraspecific variation, and covariation) per site and treatment were calculated as proportions of total variation.

To examine the variability in TA functional traits driven by environmental filtering and niche differentiation in ambient and WLD conditions, we first used violin plots to compare community-weighted trait distributions and assess differences in distribution shape as potential signals of adaptation to WLD. To select the significant traits (p-value < 0.05), we used the results of multivariate analysis of variance (MANOVA) from the same data reported in Kuuri-Riutta [25]. Skewness and kurtosis were quantified for each of these traits in each study area. As most distributions were non-Gaussian, we calculated the deviations in WLD study areas relative to the mean of all control study areas (% difference) to allow comparisons between treatments [36].

To further quantify the effects of environmental filtering and niche differentiation on size-related traits, we used trait-based null models for TA biovolume and aperture size. We constructed 999 randomly generated communities (null expectations) for each trait across the 53 sample plots using a two-step randomization process. First, species were randomly selected from the total species pool (equiprobable selection) that comprised all identified species at the site level while preserving observed species richness and relative abundances to maintain ecological relevance (e.g. avoiding rare species becoming dominant). Second, we isolate the traits effects by randomly allocating a plot-wise mean trait value to each selected species. Decisions on how to build and test the null models were based on Perronne [37, and references therein]. For each plot, four functional metrics were calculated using the observed data and compared with the corresponding null expectations. To detect environmental filtering, we compared the mean, range of traits, and mean pairwise trait distance (MTD), and to detect niche differentiation, we compared the coefficient of variation of the nearest-neighbor distance between traits (cv_nnd) [37], and references therein. We used Wilcoxon signed-rank test (non-parametric test to compare paired samples from two treatments [38]) to evaluate the deviation of the observed functional metrics from their null expectations across sites. Tests for means were two-tailed in control study areas (allowing shifts in either direction due to environmental filtering) and one-tailed in WLD study areas (expecting downward shifts from treatment). Range, cv_nnd, and MTD tests were one-tailed in control and WLD study areas (with directional shifts expected from environmental filtering and niche differentiation). Secondly, to quantify the strength of the effect of environmental filtering and niche differentiation, we calculated the standardized effect size (SES) as the difference between the observed values and the null expectation mean value divided by the standard deviation of the null expectation [37, and references therein].

To examine the variability in TA functional structure between ambient and WLD conditions, we used three beta functional diversity indices as proposed by Ricotta & Pavoine [39]: ‘beta redundancy’ (different species supporting similar functions between communities), ‘functional dissimilarity’ (functional divergence among species), and ‘taxonomic similarity’ (shared species among communities). We used *adiv* package [40] for the calculations and a ternary diagram from *ade4* package [41] for illustration. To test functional structure differences between sites and treatments, we used db-MANOVA from *PERMANOVA* package [42] and betadisper from *vegan* package [34]. Individual indexes were tested using two-way permutational ANOVA from package *lmperm* [43].

## Results

### Variability in Functional Compositions of Testate Amoebae Between Ambient and WLD Conditions

**Fig. 1:**
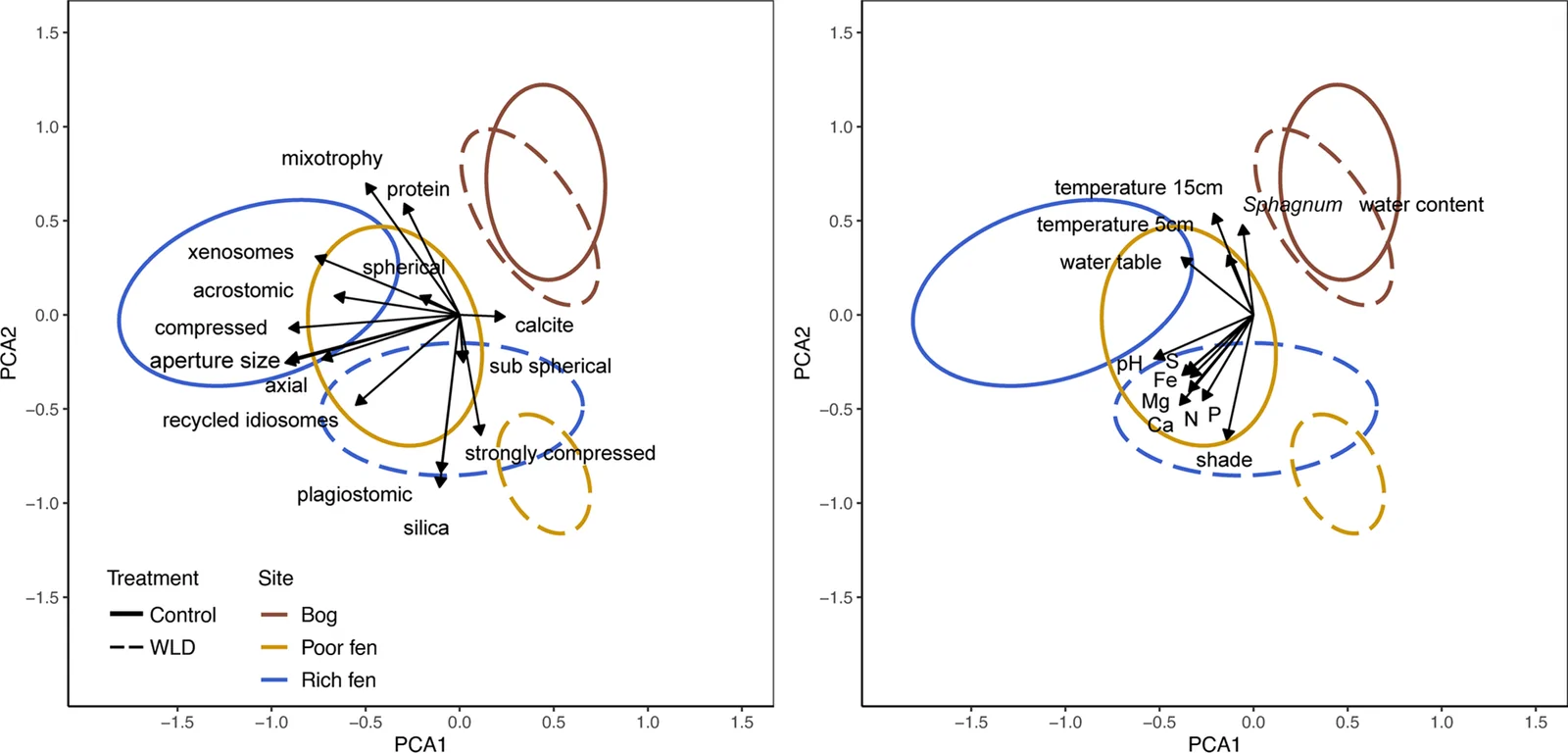
PCA of TA community functional traits. Left biplot shows the importance of different functional traits for PC axes 1 (PCA1) and 2 (PCA2); right biplot shows correlations of environmental variables. Ellipses indicate sample positions by treatment and site within the ordination space

TA functional traits differed among sites and between treatments (Fig. 1; Table S1). Under ambient conditions, the bog-rich fen gradient was expressed as shifts from smaller to larger biovolume and aperture size, from higher proportion of mixotrophs and proteinaceous tests to assemblages dominated by acrostomic apertures, compressed and recycled idiosomic tests.

WLD reshaped TA assemblages across sites by reducing size-related traits (biovolume and aperture) and mixotrophy, shifting apertures positions from axial to plagiostomic, and redistributing test materials from proteinaceous and xenosomic towards siliceous; with greater treatment differences in fens than in bog (Table S1). Consistently, WLD significantly predicted variation in the community-weighted size traits, explaining 22% of biovolume variation and 17% of aperture size variation (Table S2). This WLD-induced size reduction was mainly driven by species turnover (15% and 14%, respectively), with minor contribution from intraspecific variation (0.7% and 0.2%), and a positive covariation effect between both mechanisms (Table S2). When the total variation in the dataset (site fertility and WLD) was considered, the dominance of species turnover was further emphasized (85% and 73%), while the contribution of intraspecific variation slightly increased (2% and 5%) (Table S2).

### Variability in Functional Traits of Testate Amoebae Driven by Environmental Filtering and Niche Differentiation in Ambient and WLD Conditions

**Fig. 2:**
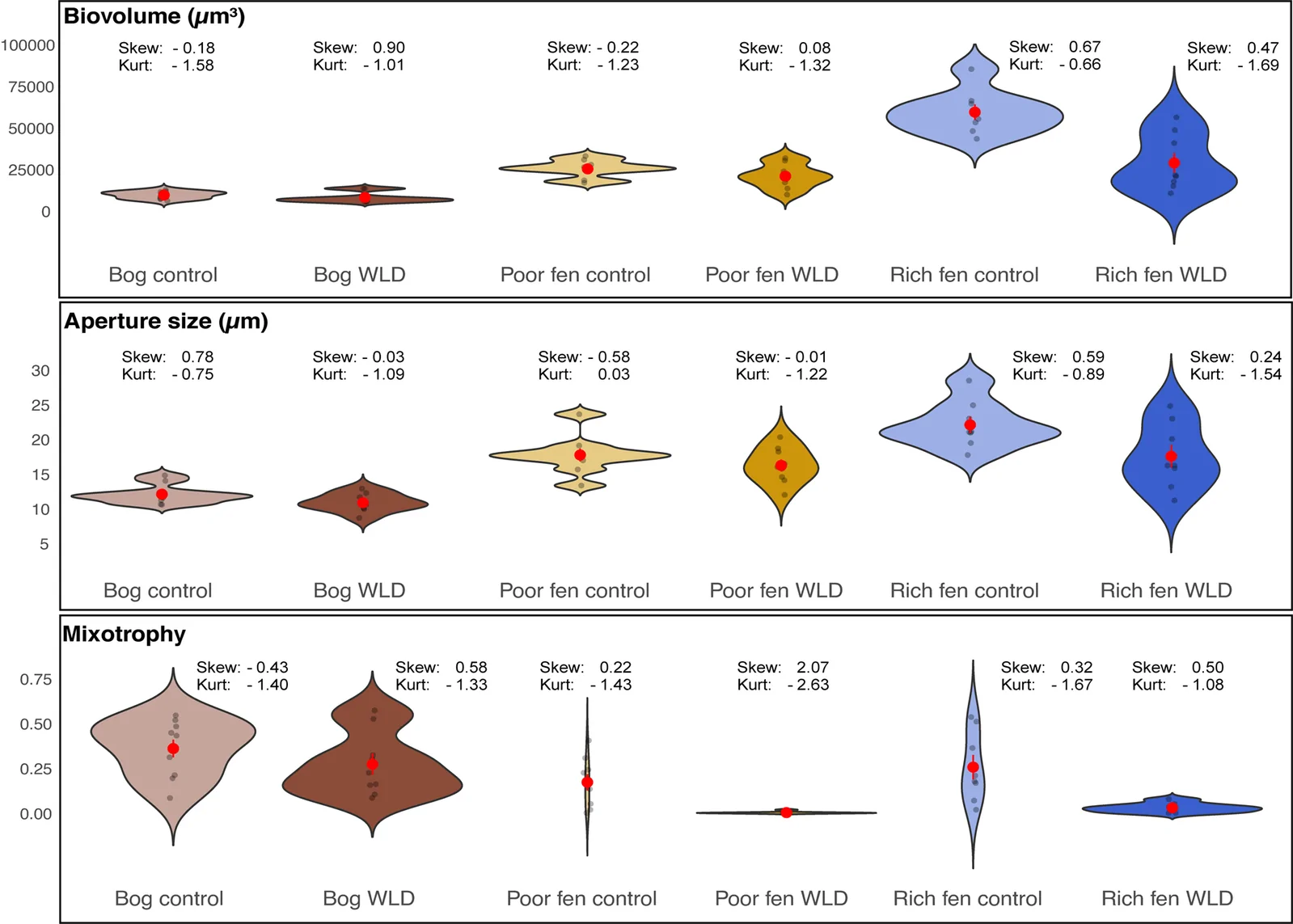
Violin plots showing community-weighted distributions for biovolume, aperture size, and mixotrophy in TA communities. Distributions differed significantly between treatments and site-treatment interactions (*p* < 0.05; MANOVA tests). Observations are overlaid as semitransparent points with a red dot for the mean point

Skewness and kurtosis of community-weighted functional trait distributions were small (between − 2 and 2, i.e., within the range suggested by Gross [9]) in both WLD and control study areas (Fig. 2, Fig. S1a - b). Deviations showed no notable differences (< 200, threshold suggested by Wieczynski [36]) between treatments, with exception of mixotrophy in the poor fen WLD area (Fig. S2).

**Fig. 3:**
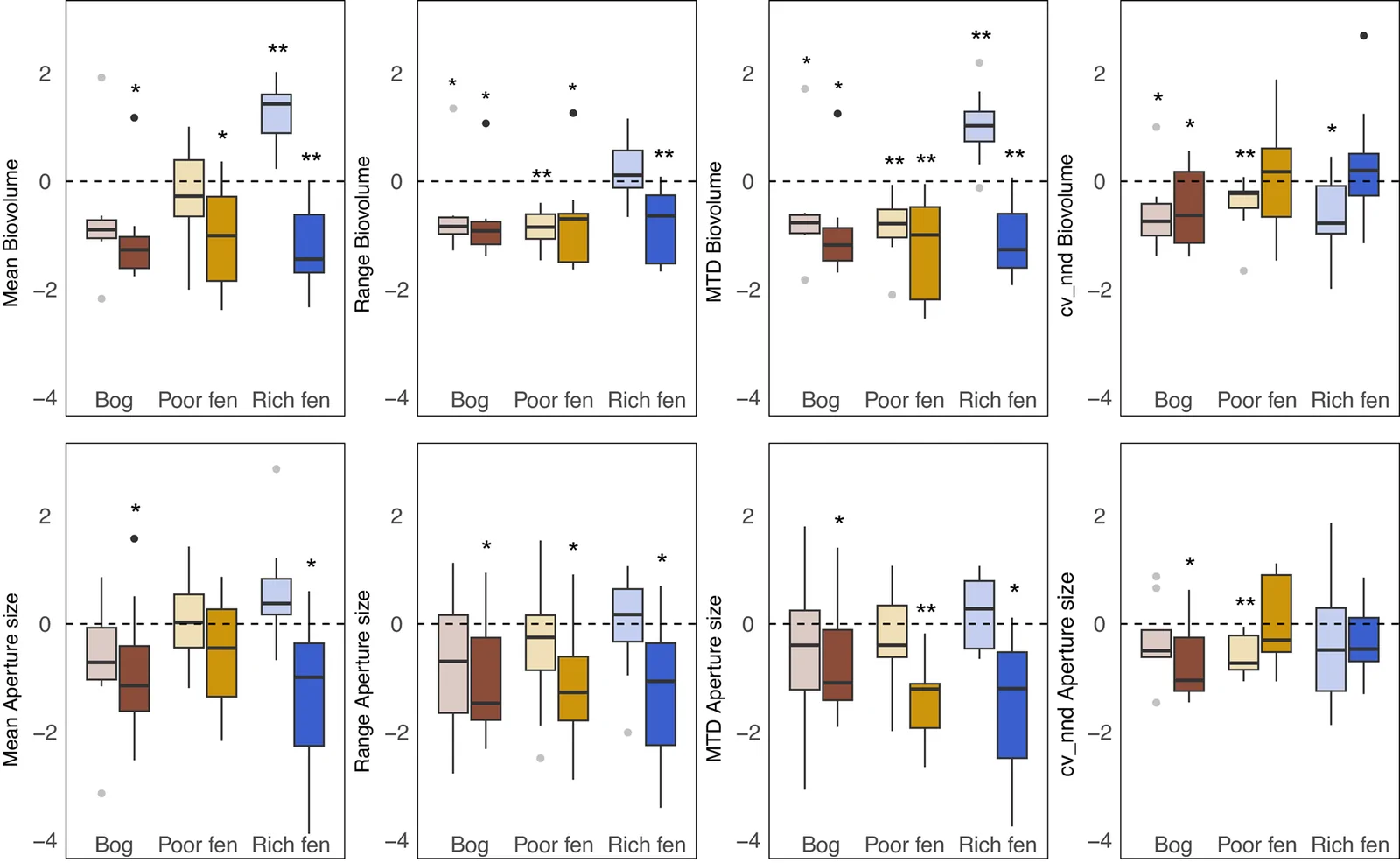
Null model comparisons for size traits (biovolume and aperture size) across sites and treatments (control vs. WLD) using four functional metrics: mean, range, mean pairwise trait distance (MTD), and coefficient of variation of nearest-neighbour distance (cv_nnd). Stars denote Wilcoxon signed-rank test significance (*p < 0.05, **p < 0.01) of observed vs. random distributions. Effect sizes use a standardized metric (SES = (observed - null mean)/null SD); see Materials and Methods for details of the tested hypotheses. Darker colors indicate the WLD treatment

Comparing size-related trait distributions with null distributions revealed stronger signals of deterministic processes under WLD than under ambient conditions. In control areas, niche differentiation dominated in the rich fen and environmental filtering in the poor fen. In the bog control area, the two processes were equally strong. In the rich fen and in the bog, niche differentiation was detected in biovolume but not in aperture size, whereas weak niche differentiation was detected by both traits in the poor fen. Environmental filtering was detected by biovolume in the poor fen and in the bog (Fig. 3, Table S3).

Under WLD conditions, environmental filtering was the dominant deterministic process, detected by both biovolume and aperture size, with stronger effect sizes than under ambient conditions. The strongest filtering was observed in the rich fen. Niche differentiation was observed in both traits only in the bog WLD area (Fig. 3, Table S3).

### Variability in Functional Structure of Testate Amoebae Between Ambient and WLD Conditions

**Fig. 4:**
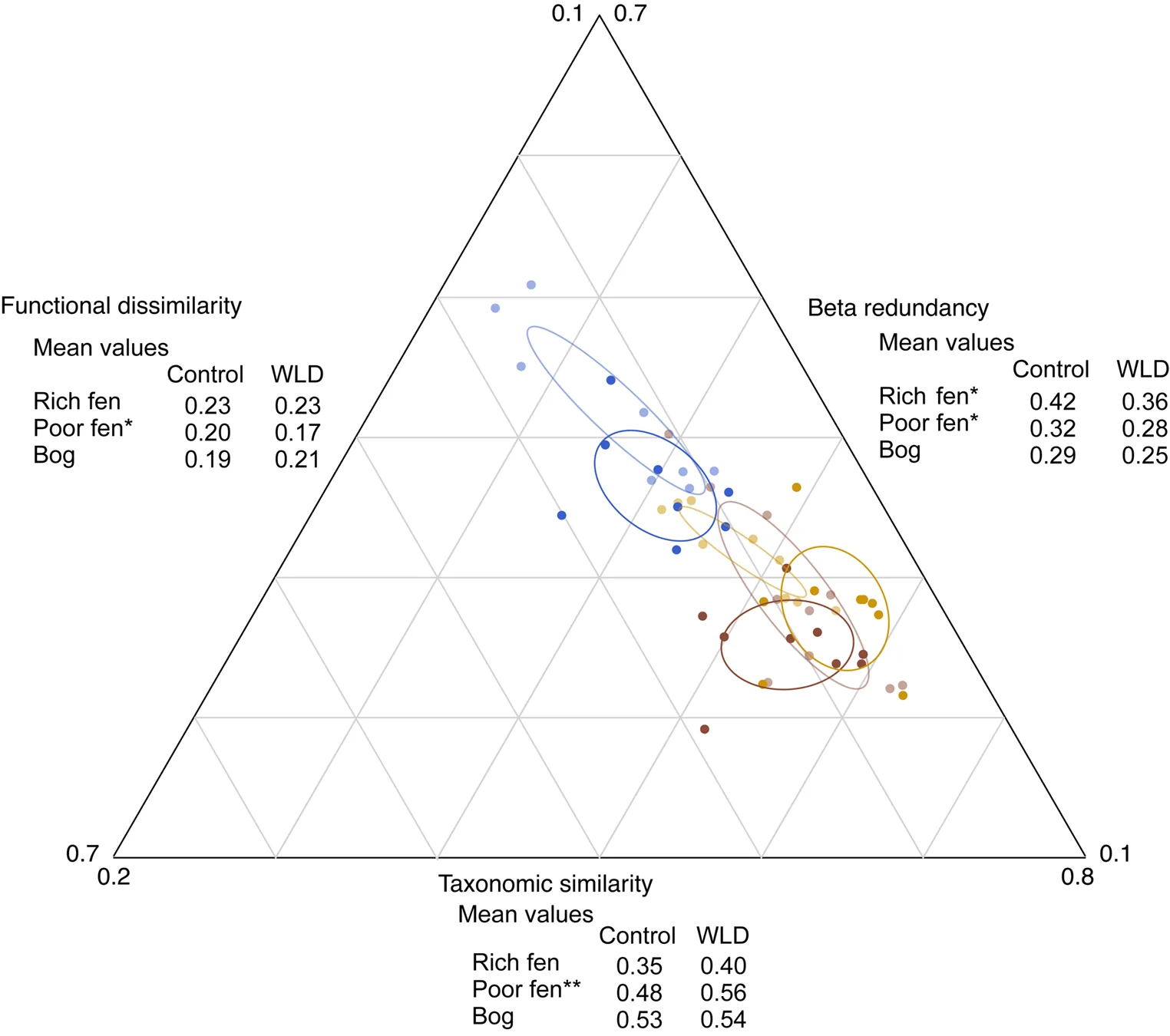
Triangle plot showing the functional structures and differences in indexes (beta redundancy, functional dissimilarity, and taxonomic similarity) between treatments (control vs. WLD). Differences were tested with permutational two-way ANOVA (*p < 0.05, **p < 0.01)

The functional structure of TA communities was measured as beta redundancy, functional dissimilarity, and taxonomic similarity. In control areas, it differed significantly between rich fen and bog and between rich fen and poor fen. TA communities in poor fen and bog shared similar functional structures, both in control and WLD areas (Fig. 4, Table S4). The test for homogeneity of variance showed no significant pairwise differences between the study areas, therefore differences/similarities in functional structure derived from their average compositions (Fig. 4, Table S4).

Beta redundancy was significantly lowered in both fen WLD areas. Additionally, functional dissimilarity decreased and taxonomic similarity increased in the poor fen WLD. On the contrary, no significant differences were observed between control and WLD in the bog (Fig. 4). When considering beta redundancy, taxonomic similarity, and functional dissimilarity together, only poor fen showed significant differences between WLD and control.

## Discussion

We applied functional traits to investigate the mechanisms that have shaped TA communities along a bog-rich fen gradient and assessed how two decades of experimental water level drawdown altered their functionality. The long-term drying acted as an environmental filter that produced drought-adapted functional profiles driven mainly by species turnover. The differences in trait-mediated stability among sites reflected variation in functional thresholds. Together, our results show that functional traits play a central role in stability; however, the strength of the functional buffering effect depends on the disturbance and the local site characteristics that modify the trait-mediated community responses and resilience and resistance capacities.

### Differences in Abiotic and Biotic Pressure on TA Communities between Ambient and WLD Conditions

Our first hypothesis, that TA communities are more strongly filtered by environment under drying conditions than under ambient conditions, was supported across sites as the strength of environmental filtering increased in WLD areas. The generally small skewness and kurtosis in our dataset indicate that TA traits are not currently exposed to strong pressure even in WLD areas, suggesting that communities have largely adapted to current biotic and abiotic conditions [9]. This is likely due to tree encroachment – that fundamentally altered local microclimates and resource environments – occurring already for more than a decade in fen WLDs [23]. Comparatively, the rapid life cycle of TA allows communities to respond to environmental changes within months to a few years [19]. In line with the dynamic nature of community assembly, where processes vary in effect and strength overtime [44], WLD communities shifted from stochastic dominance toward more deterministic processes. This shift allowed us to identify the deterministic processes and quantify the strength of their influence.

Under ambient conditions, niche differentiation was the primary mechanism structuring TA communities in the rich fen control. This highlights the strong effect of biotic interactions in resource-rich habitats that allow diverse species with contrasting ecological strategies to reach stable competitive differentiation, thus promoting coexistence [45]. In contrast, in the bog control, where water and nutrient supply depend on precipitation, niche differentiation and environmental filtering were equally strong. Under such resource-limited conditions, species are driven to use the limited resources differently to avoid direct competition (i.e., resource partitioning) [46]. In the poor fen control, both processes were detected, but environmental filtering had a stronger effect compared to niche differentiation. Because the poor fen represents resource-limited conditions among fen habitats, TA communities there likely have undergone strong selection for traits improving tolerance to nutrient-poor and acidic conditions. This has led to species occupying distinct niches that reduce overlap and enable efficient resource partitioning among coexisting species [45, 46].

Under experimental drying (i.e., dry and more shaded conditions), community assembly across all WLD areas was dominated by a strong environmental filtering, which consistently reduced biovolume and aperture size of TA communities. Small individuals have multiple competitive advantages in thin water films around *Sphagnum* stems and leaves: they have a reduced risk of desiccation [19, 31], can reproduce faster, require smaller living space [18], and recolonize faster from adjacent areas [47]. In the bog WLD area specifically, environmental filtering intensified niche differentiation for both size traits. This pattern aligns with previous findings that in resource-limited habitats, environmental changes impose additional pressure on community dynamics [48], leading to stronger trait-based structuring of TA communities. Moreover, decreasing size under drying reflects a shift towards lower trophic levels among predators [42]. Such size restructuring, along with shifts in vegetation from *Sphagnum-*dominated peatlands to forest, can alter the diversity and structure of the microbial food web [21, 48].

Aperture size and biovolume reflected contrasting roles in mediating community stability and responses to environmental pressure. Biovolume variation was mainly driven by environmental change and species turnover, while aperture size variation was strongly associated with niche differentiation and higher intraspecific variation. Consistent with this, Bobrov & Mazei [49] reported that aperture size showed the greatest variability among size traits across habitats and can change independently of overall test size. This likely reflects the functional importance of the TA aperture as the main interface with the environment, as it is used in feeding, locomotion, and environmental sensing [18, and references therein]. These contrasting responses highlight how trait-specific strategies underpin community stability, with both size traits variation contributing to resilience, and aperture variation promoting resistance under the drying pressure.

## Functional Stability of Testate Amoeba Community Mediated by Functional Redundancy

Our second hypothesis - that functionally redundant TA communities are less vulnerable to climate-induced drying - was supported in the fens. In the rich fen, functional redundancy peaked together with functional stability, whereas in the poor fen, where redundancy was low, drying led to functional turnover. However, the bog remained functionally stable despite low redundancy.

In the rich fen, despite the strongest WLD-induced species turnover, the overall functional structure of TA did not differ between treatments. Our results suggest that the taxonomically diverse [25] and functionally redundant TA community in the rich fen was functionally resilient, i.e., the species lost due to drying were replaced by functionally similar species. For example, mixotrophic *Amphitrema stenostoma* and *Amphitrema wrightianum* were lost from WLD due to their hydrological sensitivity [50], but mixotrophy was maintained by the remaining three mixotrophic species. These results reflect the functional insurance effect, according to which the presence of numerous species performing similar functions promotes the functional stability of an ecosystem against disturbances [11, 13]. Furthermore, diversity may favour the emergence of novel interactions among species, including complementarity mechanisms [51].

In the bog, species turnover was the smallest, and functional structure did not differ significantly between treatments despite low functional redundancy. Therefore, functional stability in the bog relied on resistance. In unevenly structured communities, such as in the bog, functional stability depends on the competitive strength and resistance of the dominant species [12, 13], whose traits were here adapted to dry and nutrient-poor conditions, e.g., small test sizes [30, 50]. Thus, the experimental water level drawdown did not exceed the environmental resistance threshold of the prevailing trait composition. Moreover, the dominance of mixotrophic *Archerella flavum* suggests that the pressure from WLD was weaker than in the fens, given that mixotrophs are highly sensitive to habitat changes [29, 52]. This likely reflects the smaller shifts in vegetation structure and abiotic conditions (e.g., shading intensity). Additionally, *Sphagnum* mosses may have buffered water table decline through capillarity [23, 24] and through decreased height growth, thereby maintaining stable microhabitat conditions. This suggests that a more severe disturbance, such as forestry drainage, would be required to produce detectable functional shifts in these bog soil biotic communities [11, and references therein].

The poor fen was the only site where taxonomic and functional differences were coupled, indicating that WLD-induced species loss resulted in functional loss. For instance, carbon-fixation function was almost totally lost along with mixotrophic species. Under ambient conditions in the poor fen, TA communities resembled the functional structure of those of the bog reflecting a limited functional insurance effect. However, the trait composition was adapted to wetter conditions than in the bog (i.e., larger test sizes, more exposed test apertures), making the community inherently more sensitive to drying. Additionally, the poor fen WLD faced more drastic ecosystem changes than the other two WLD areas, including increased nutrient concentrations, higher shading intensity, and substantial replacement of *Sphagnum* by vascular plants [23, 24] – shifts known to affect TA communities [51, 52]. This triggered competitive exclusion of moisture and light-demanding TA. Furthermore, species and functional turnover narrowed the functional space, as unique species occupied substantially less niche volume than under ambient conditions, weakening community stability to further disturbances. Thus, as climate warming intensifies drying, the resilience of TA — and the ecosystem functions they mediate — is increasingly at risk of being compromised [11, 51]. Altogether, our findings provide mechanistic insights into how trait-mediated soil biodiversity supports ecosystem stability and resilience, offering predictive understanding for peatland responses to global change. In line with “precision ecology” [53], effective climate adaptation and mitigation of peatlands will require habitat-specific strategies that enhance their functional resilience.

## Supplementary Information

Below is the link to the electronic supplementary material.

Supplementary Material 1 (DOCX 889 KB)

## Data Availability

The microbial data are openly available in Zenodo (10.5281/zenodo.17940365). The environmental data from Kokkonen [54] are stored in Pangaea Data Library (10.1594/PANGAEA.904256), and those from Kuuri-Riutta et al. [55, 56] are storedin IDA research data storage service (10.23729/fd-bad048dd-eda3-380f-915a-580ccd150ec2).
