# Supplementary material for: Contrasting Trait-Mediated Mechanisms Shape Peatland Testate Amoeba Communities Under Long-Term Drying Across Fen-Bog Gradient
Source: Microb Ecol. 2026 Jun 3;89(1):147. doi: 10.1007/s00248-026-02796-1 (PMC13372875; doi:10.1007/s00248-026-02796-1)
Supplement: Supplementary file 1 — Supplementary Material 1 (DOCX 889 KB) [file 248_2026_2796_MOESM1_ESM.docx]

**Supplementary information**

**Contrasting trait-mediated mechanisms shape testate amoeba communities under long-term drying across boreal peatlands**

**Microbial Ecology**

Brunella Palacios Ganoza, Olivia Kuuri-Riutta, Anna M. Laine, Minna M. Väliranta, Edward A. D. Mitchell, Eeva-Stiina Tuittila

Corresponding author: Brunella Palacios Ganoza, School of Forest Sciences, University of Eastern Finland, Joensuu, Finland, [brunella.palacios.ganoza@uef.fi](mailto:brunella.palacios.ganoza@uef.fi)

**Table S1** Results of the multivariate analysis of variance (MANOVA) testing site (rich fen, poor fen, and bog) and treatment (water level drawdown) differences on functional traits. P-values refer to unadjusted p-values.

|  | | Site | | Treatment | | Site*Treatment | |
| --- | --- | --- | --- | --- | --- | --- | --- |
|  |  | dev | p-value | dev | p-value | dev | p-value |
| Aperture size | | 857.03 | 0.001 | 198.30 | 0.001 | 140.50 | 0.013 |
| Biovolume | | 65782.96 | 0.001 | 22463.46 | 0.001 | 6486.07 | 0.001 |
| Mixotrophy | | 13.31 | 0.031 | 18.34 | 0.001 | 28.08 | 0.001 |
| Aperture position | Axial | 3.17 | 0.183 | 4.09 | 0.02 | 4.81 | 0.04 |
|  | Acrostomic | 18.22 | 0.005 | 1.72 | 0.215 | 0.04 | 0.973 |
|  | Plagiostomic | 14.85 | 0.013 | 7.69 | 0.019 | 0.25 | 0.92 |
| Test compression | Spherical | 2.59 | 0.311 | 3.19 | 0.096 | 5.32 | 0.066 |
|  | Sub spherical | 3.32 | 0.221 | 0.02 | 0.876 | 2.75 | 0.259 |
|  | Compressed | 36.66 | 0.001 | 2.83 | 0.079 | 6.83 | 0.017 |
|  | Strongly compressed | 7.89 | 0.007 | 0.07 | 0.731 | 0.15 | 0.877 |
| Test material | Protein | 12.66 | 0.016 | 11.20 | 0.003 | 21.20 | 0.002 |
|  | Silica | 25.86 | 0.006 | 8.76 | 0.016 | 1.51 | 0.61 |
|  | Silica + org | 7.94 | 0.004 | 0.01 | 0.84 | 1.40 | 0.156 |
|  | Calcite | 4.08 | 0.025 | 0.01 | 0.892 | 0.90 | 0.575 |
|  | Recycled idiosomes | 65.32 | 0.001 | 0.08 | 0.755 | 1.97 | 0.316 |
|  | Xenosomes | 6.64 | 0.11 | 6.20 | 0.029 | 1.49 | 0.509 |

**Table S2** Variance partitioning for community weighted (CWM) aperture size and biovolume with site fertility, treatment (water level drawdown), and their interaction as explanatory variables at community level. The different levels of statistical significance are expressed as p < 0.05*, p < 0.01**, p < 0.001***.

| Biovolume | Sum of squares | | | |
| --- | --- | --- | --- | --- |
| Explanatory variables | Species turnover | Intraspecific variation | Covariation | Species turnover and Intraspecific variation |
|  |  |  |  |  |
| Site fertility (SF) | 6 216 377 825*** | 22 956 341** | 755 527 065 | 6 994 861 231*** |
| Treatment | 2 746 075 528*** | 127 331 795*** | 1 182 645 721 | 4 056 053 044*** |
| SF*Treatment | 1 728 467 210*** | 72 583 409*** | 708 401 135 | 2 509 451 754*** |
| Residual variance | 5 206 264 534 | 130 394 939 | -294 175 411 | 5 042 484 062 |
|  | | | | |
| Biovolume variation | Species turnover  (%) | Intraspecific variation (%) | Covariation (%) | Species turnover and Intraspecific variation (%) |
| Site fertility (SF) | 33.42 | 0.12 | 4.06 | 37.60 |
| Treatment | 14.76 | 0.68 | 6.36 | 21.80 |
| SF*Treatment | 9.29 | 0.39 | 3.81 | 13.49 |
| Residual variance | 27.99 | 0.70 | -1.58 | 27.11 |
| Total | 85.46 | 1.90 | 12.65 | 100.00 |

| Aperture size | Sum of squares | | | |
| --- | --- | --- | --- | --- |
| Explanatory variables | Species turnover | Intraspecific variation | Covariation | Species turnover and Intraspecific variation |
|  |  |  |  |  |
| Site fertility (SF) | 291.75*** | 11.48*** | 115.78 | 419.01*** |
| Treatment | 154.64*** | 2.15 | 36.49 | 193.28*** |
| SF*Treatment | 33.02* | 0.74 | 9.88 | 43.64* |
| Residual variance | 327.37 | 42.69 | 82.56 | 452.62 |
|  | | | | |
| Aperture size variation | Species turnover (%) | Intraspecific variation (%) | Covariation  (%) | Species turnover and Intraspecific variation (%) |
| Site fertility (SF) | 26.32 | 1.04 | 10.44 | 37.80 |
| Treatment | 13.95 | 0.19 | 3.29 | 17.44 |
| SF*Treatment | 2.98 | 0.07 | 0.89 | 3.94 |
| Residual variance | 29.53 | 3.85 | 7.45 | 40.83 |
| Total | 72.78 | 5.15 | 22.07 | 100.00 |


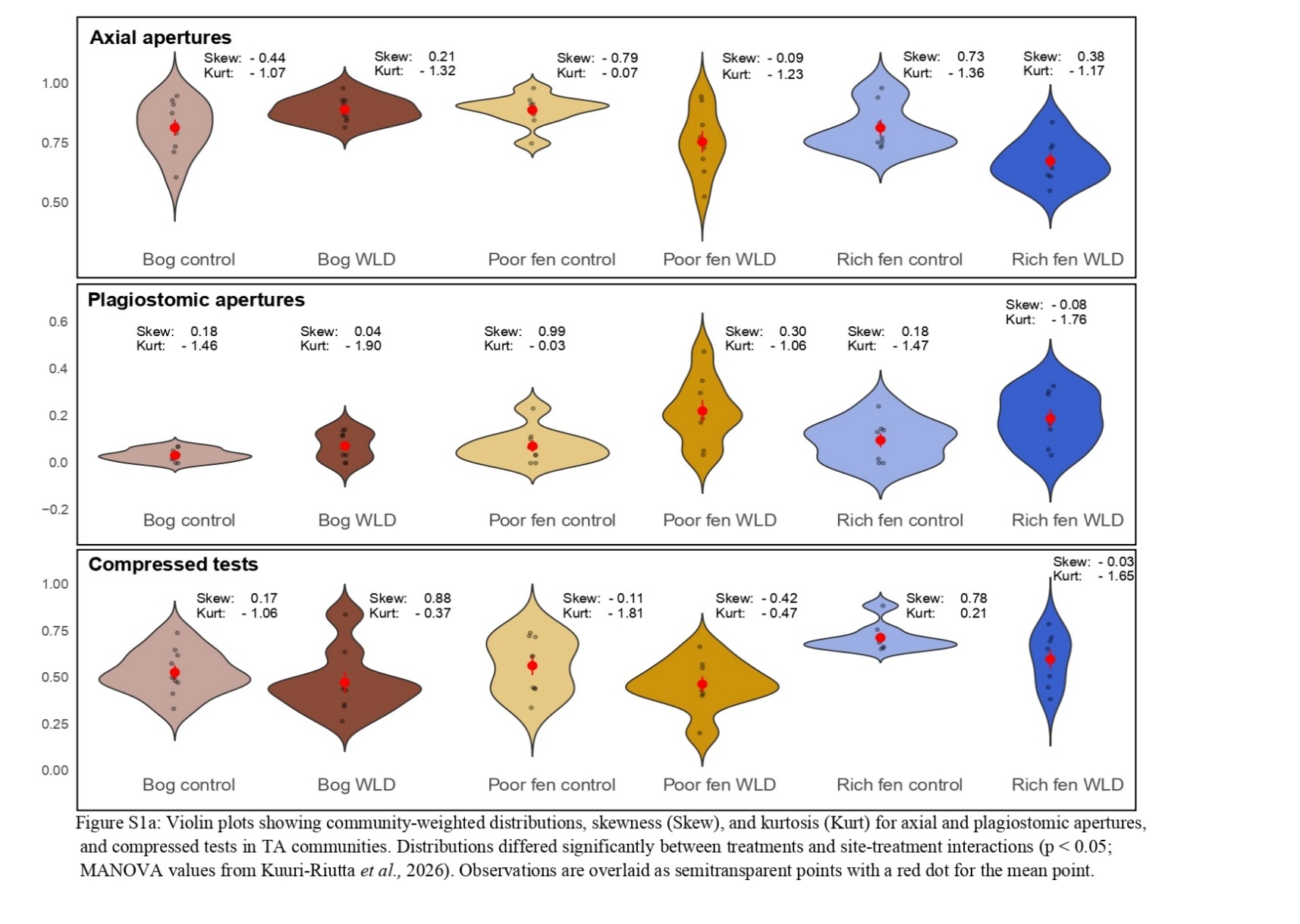


**Fig. S1 (a)** Violin plots showing community-weighted distributions, skewness (Skew), and kurtosis (Kurt) for axial and plagiostomic apertures, and compressed tests in TA communities. Distributions differed significantly between treatments and site-treatment interactions (p < 0.05; MANOVA values from Kuuri-Riutta *et al.,* 2026). Observations are overlaid as semitransparent points with a red dot for the mean point.

**
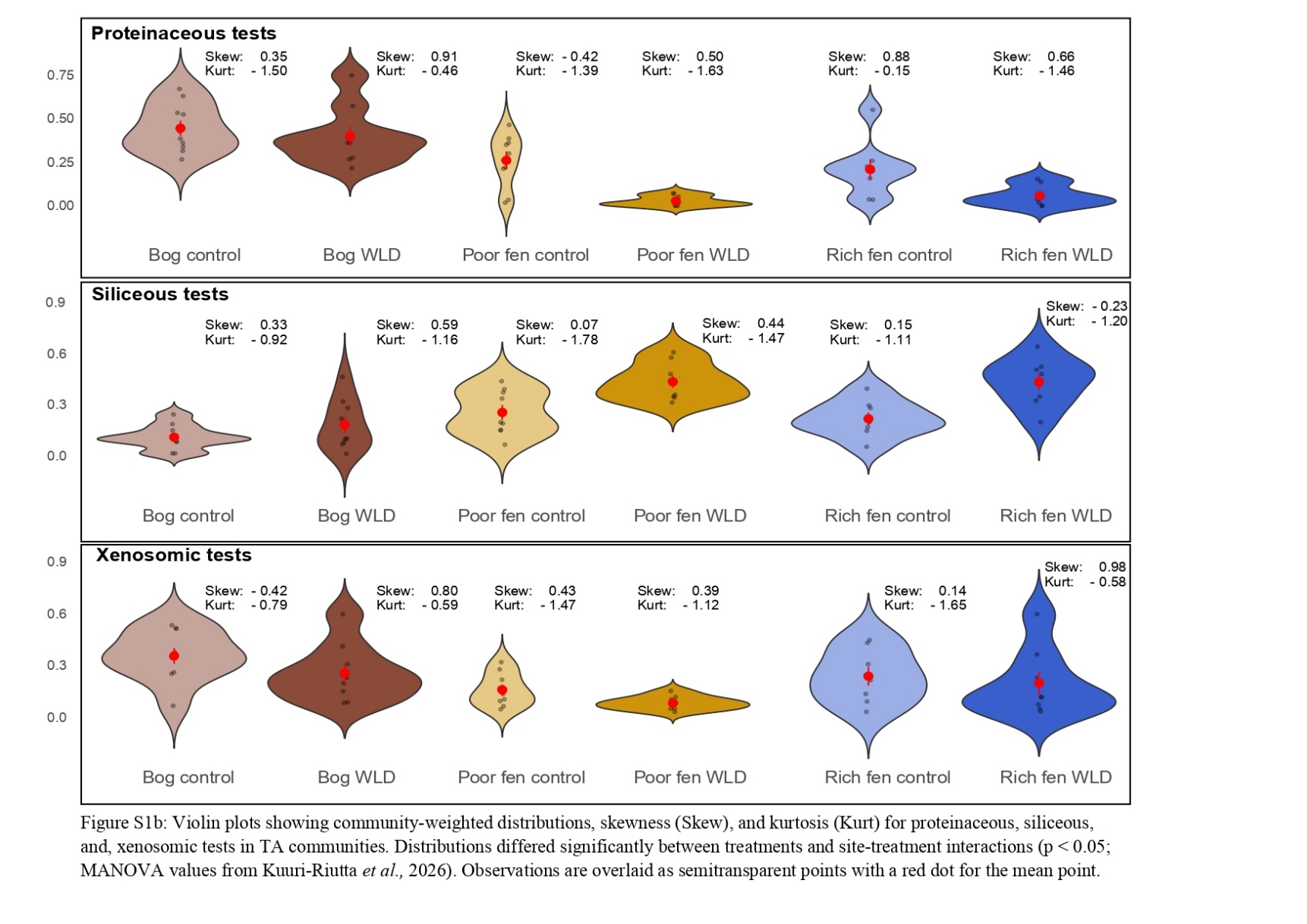
**

**
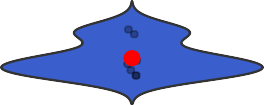
Fig. S1 (b)** Violin plots showing community-weighted distributions, skewness (Skew), and kurtosis (Kurt) for proteinaceous, siliceous, and xenosomic tests in TA communities. Distributions differed significantly between treatments and site-treatment interactions (p < 0.05; MANOVA values from Kuuri-Riutta et al., 2026). Observations are overlaid as semitransparent points with a red dot for the mean point.

**
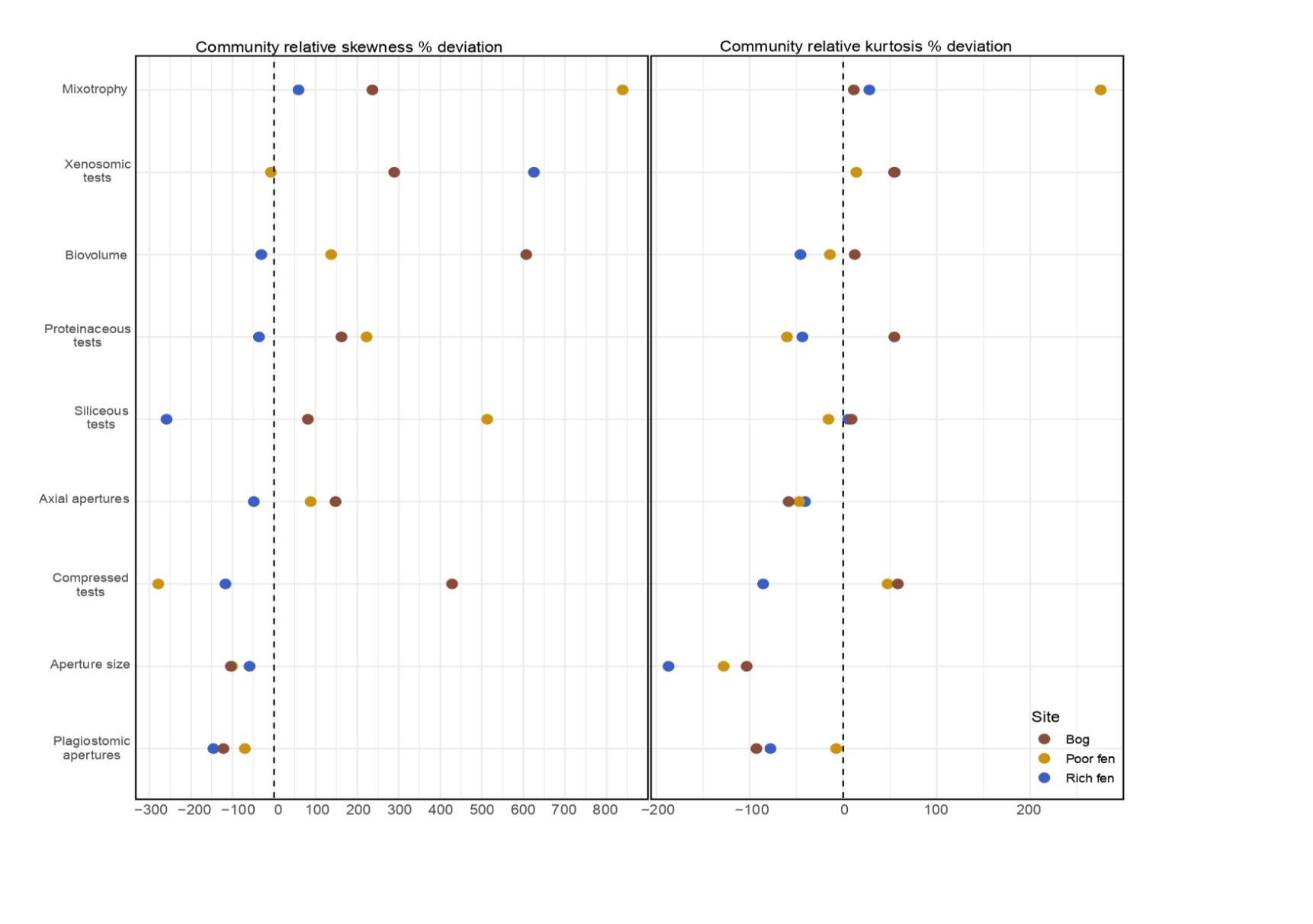
**

**Fig. S2** Community relative skewness and kurtosis expressed as the percentage of deviation of water level drawdown (WLD) areas from ambient values (control) for each functional trait.

**Table S3** Nonparametric Wilcoxon signed-rank tests for significant differences between null distributions and observed data for biovolume and aperture size between treatments. For environmental filtering, two-tailed tests were applied to control area means, one- tailed to water level drawdown (WLD) area means, and one-tailed to range and mean pairwise trait distance (MTD). For niche differentiation, a one-tailed test was used for the coefficient of variation of nearest neighbor distance (cv_nnd) in both control and WLD areas.

| Functional trait | Functional metric | Wilcoxon p-value | | | | | |
| --- | --- | --- | --- | --- | --- | --- | --- |
|  |  | Rich fen | | Poor fen | | Bog | |
|  |  | Control | WLD | Control | WLD | Control | WLD |
| Biovolume | Mean | 0.008 | 0.004 | 0.49 | 0.01 | 0.08 | 0.01 |
|  | Range | 0.84 | 0.008 | 0.002 | 0.02 | 0.04 | 0.03 |
|  | cv_nnd | 0.03 | 0.84 | 0.004 | 0.59 | 0.02 | 0.04 |
|  | MTD | 0.008 | 0.008 | 0.002 | 0.002 | 0.04 | 0.01 |
| Aperture size | Mean | 0.08 | 0.02 | 0.50 | 0.10 | 0.06 | 0.05 |
|  | Range | 0.62 | 0.02 | 0.24 | 0.02 | 0.07 | 0.01 |
|  | cv_nnd | 0.27 | 0.12 | 0.002 | 0.71 | 0.16 | 0.01 |
|  | MTD | 0.77 | 0.02 | 0.21 | 0.002 | 0.19 | 0.05 |

**Table S4** Site pair-wise comparison of functional structure variation and dispersion between sites (Rich fen, poor fen, and bog) and treatments (control and WLD). Compositional variation was tested using permutational multivariate ANOVA based on distances and homogeneity of dispersion to group centroids with Levene´s test based on distances. WLD = water level drawdown.

| Site pairwise-comparisons | Functional structure | | | |
| --- | --- | --- | --- | --- |
|  | Compositional variation | | Dispersion (homogeneity) | |
|  | F | p-value adj | F | p-value adj |
| Rich fen control – Poor fen control | 15.31 | < 0.001 | 1.36 | 0.26 |
| Poor fen control – Bog control | 2.82 | 0.10 | 0.69 | 0.42 |
| Bog control – Rich fen control | 22.80 | < 0.001 | 0.13 | 0.72 |
| Rich fen WLD – Poor fen WLD | 34.62 | < 0.0001 | 0.09 | 0.77 |
| Poor fen WLD – Bog WLD | 2.90 | 0.07 | 0.06 | 0.82 |
| Bog WLD – Rich fen WLD | 30.33 | < 0.0001 | 0.01 | 0.91 |
| Rich fen control – Rich fen WLD | 2.69 | 0.11 | 1.25 | 0.28 |
| Poor fen control – Poor fen WLD | 9.47 | 0.004 | 0.11 | 0.74 |
| Bog control – Bog WLD | 1.28 | 0.29 | 0.97 | 0.34 |
